# Obtaining Valid Compatibility Intervals for Sequence Symmetry Analyses Utilizing Active Comparators: A Simulation Study

**DOI:** 10.1002/pds.70160

**Published:** 2025-05-20

**Authors:** Martin Torp Rahbek, Jesper Hallas, Lars Christian Lund

**Affiliations:** ^1^ Clinical Pharmacology, Pharmacy and Environmental Medicine University of Southern Denmark Odense Denmark

**Keywords:** active comparator, compatibility intervals, sequence symmetry analysis, simulation study

## Abstract

**Purpose:**

To compare different methods of estimating 95% compatibility intervals (CIs) for the sequence ratio (SR) when performing a sequence symmetry analysis using an active comparator to reduce the risk of time‐varying confounding.

**Methods:**

We conducted a simulation study, where we simulated drug‐outcome and outcome‐drug sequences for a drug of interest and a comparator drug using the binomial distribution and obtained active comparator SRs and 95% CIs. We simulated scenarios with sample sizes between 5 and 50 observed sequences for each SR, which could take values of 0.5, 1.0, or 2.0, yielding 276 scenarios that were replicated 5000 times. For each replication, we calculated 95% CIs using current recommendations based on exact CIs, the Woolf logit, Baptista‐Pike mid‐*p*, and Miettinen–Nurminen score estimator and calculated coverage for each scenario.

**Results:**

All interval estimators provided acceptable coverage when sample sizes exceeded 15, except for the current recommendation, the exact Clopper–Pearson interval. The Miettinen‐Nurminen score (coverage 0.951) and Baptista–Pike mid‐*p* interval (coverage 0.955) offered more accurate coverage than other methods. The largest divergence from 0.95 was observed for the current recommendations (coverage 0.979).

**Conclusions:**

The Miettinen–Nurminen score estimator provided the most accurate coverage for 95% CIs of active comparator SRs, especially with low sample sizes. Therefore, we recommend using the Miettinen–Nurminen score estimator for active comparator SRs.

**Summary**

- Using an active comparator in sequence symmetry analysis reduces bias from confounding by indication.
- The optimal estimator for calculating 95% compatibility intervals for active comparator sequence ratios is not known.
- The Miettinen‐Nurminen score estimator achieved the most accurate 95% compatibility interval coverage of the tested estimators.
- The Miettinen‐Nurminen score estimator provides the most accurate coverage, particularly in scenarios with low sample sizes.
- The currently recommended method for estimating compatibility intervals, showed poorer performance than other of the tested methods.

## 1 Purpose

The sequence symmetry analysis is a self‐controlled design that is robust to time‐invariant confounding and has mainly been used for the detection of drug‐safety signals in large healthcare databases [1, 2]. The effect estimate obtained from the sequence symmetry analysis is the sequence ratio (SR), which is an estimate of the incidence rate ratio that would have arisen from the underlying cohort study [3]. The SR is calculated as the number of exposure‐to‐outcome sequences divided by the number of outcome‐to‐exposure sequences. Valid compatibility intervals (CIs) [4] for the SR can be estimated based on a number of different distributions, including the normal, binomial, and beta distributions [5]. Like other self‐controlled designs, the sequence symmetry analysis is vulnerable to time‐varying confounding, including confounding by indication. This bias can be minimized by applying an active comparator to the design [6]. The resulting effect estimate is the active comparator SR and is calculated as the SR for the medication of interest divided by the SR for the comparator drug. Corresponding CIs have been recommended to be calculated based on the variance of the log‐difference between the two SRs being equal to the sum of their variances [7]. The proposed method assumes that the variance follows a normal distribution, which may not be the case when the sample size is low.

Summation of variances obtained from non‐normally distributed CIs may yield CIs with suboptimal coverage. Fortunately, other methods for interval estimation exist. If we consider the SR as the odds of an exposure‐to‐outcome sequence compared to the opposite sequence, then the active comparator SR would have the properties of an odds ratio. Previous literature has examined different methods of estimating CIs for odds ratios [8], but these have not been evaluated with SRs in mind. Therefore, we aimed to compare—in a simulation study—CIs for active comparator SRs derived according to first recommendations [6] with those derived from other methods.

## 2 Methods

We conducted a simulation study, where we simulated the number of observed drug‐outcome and outcome‐drug sequences for a drug of interest and a comparator drug using the binomial distribution. Estimates of interest were the active comparator SR and the upper and lower bounds of the 95% CI and the resulting coverage.

### 2.1 Data Generating Mechanism

We simulated a range of different scenarios where we varied the observed number of sequences for each drug and the true values of the SR for each drug. The number of sequences was between 5 and 50 with true SRs of 0.5, 1 and 2, yielding 276 different scenarios (46 number of sequences × 6 unique combinations of true SRs) each simulated 5000 times. In each replication, the number of observed exposure‐to‐outcome sequences for each drug was drawn from the binomial distribution with a given number of trials—sequences—and a probability corresponding to $\frac{SR_{\text{true}}}{1+SR_{\text{true}}}$.

### 2.2 Estimand

The estimands of interest in this simulation study are the upper and lower bounds of the 95% CI for the ratio between the SR for the drug of interest and the SR for the comparator drug.

### 2.3 Estimators

We tested several methods to estimate the bounds of the 95% CIs for the active comparator SR. First, we estimated intervals as outlined in the original description of active comparators in Self‐Controlled designs [6]. We calculated the SR for each drug and obtained 95% CIs. Then, we calculated the individual variance based on the limits of the CI (Appendix 1). Finally, we summarized the variance for the two component SRs and calculated limits for the resulting CI based on the normal distribution (Appendix 2). We tested this method with three different types of CIs for the individual SRs: the Agresti–Coull interval, the Clopper–Pearson interval, and a posterior interval based on Jeffrey's noninformative prior [5]. Second, we calculated 95% CIs that were directly based on the number of observed sequences for each drug. For this, we tested the Woolf logit (Appendix 3) [9], the Baptista–Pike mid‐*p* interval [10], and the Miettinen–Nurminen score [11].

### 2.4 Performance Measures

For each scenario and estimator, we calculated the observed coverage, Monte–Carlo standard error [12] and corresponding divergence from the desired 0.95 coverage across all 5000 replications. We also calculated the overall coverage and divergence across all scenarios and replications. Here, coverage refers to the proportion of times that the true point estimate lies within the estimated CI across repeated samples. The divergence measures the deviation of the observed coverage from the desired coverage level (e.g., 0.95). A lower divergence indicates more accurate coverage.

Simulations were conducted using R version 4.3.3 [13]. We used relevant functions from the sasLM package [14] to calculate the Miettinen–Nurminen score intervals and the ORCI package [15] to calculate Baptista–Pike mid‐*p* intervals. The source code used to conduct these analyses is available from https://gitlab.sdu.dk/lclund/active‐comparator‐sr‐ci/.

## 3 Results

**FIGURE 1:**
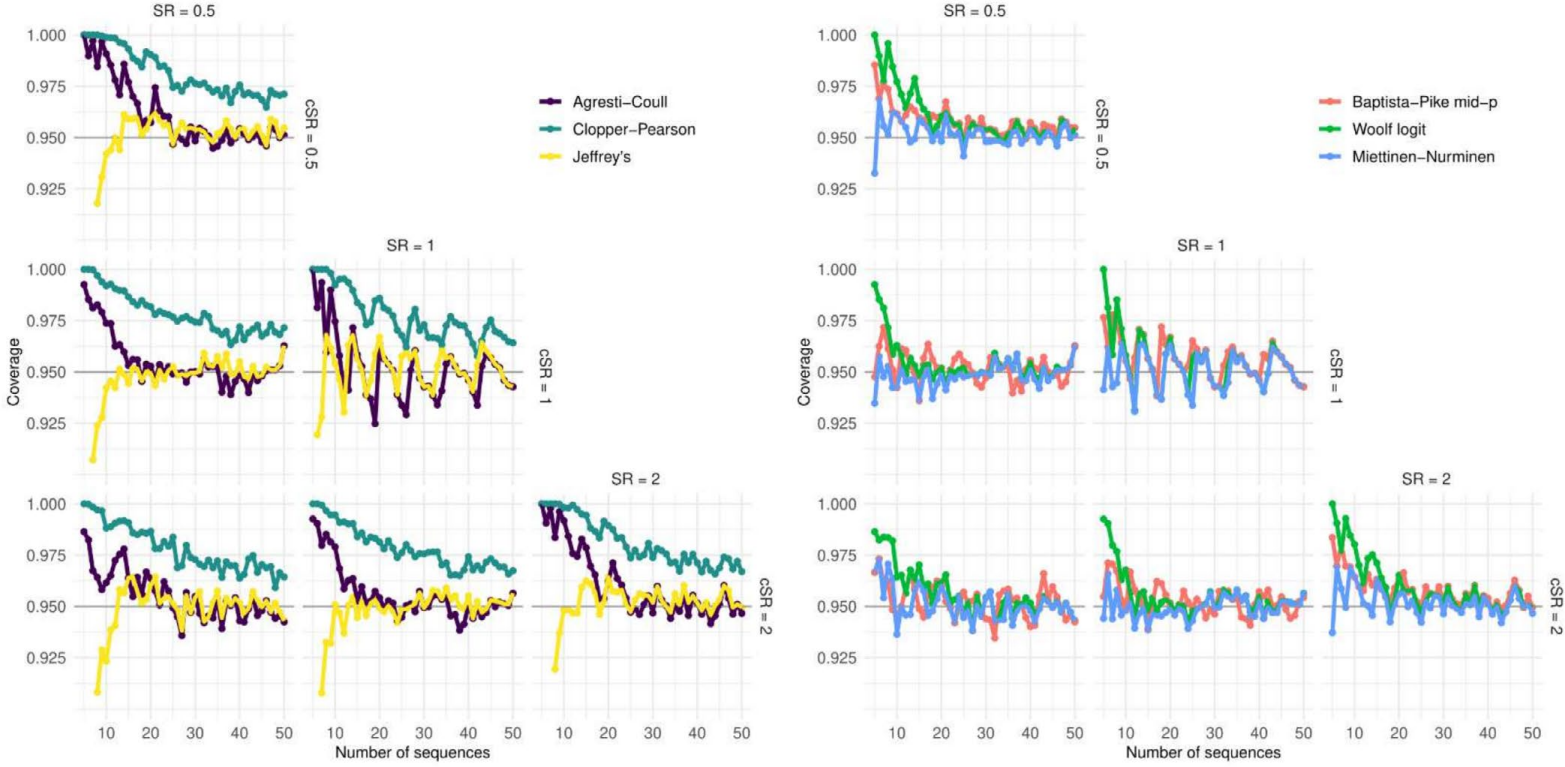
Observed coverage for interval estimators across all scenarios. *X*‐axis indicates the sample size, the *y*‐axis the observed coverage. Plots are grouped in a matrix, with columns indicating the sequence ratio of the drug of interest (0.5, 1, and 2), and rows indicating the comparator sequence ratio (0.5, 1, 2). The left panel shows the results for interval estimators based on pooled variance of interval estimators derived separately for active substance and comparator. The right panel shows interval estimators based on the observed counts directly, without intermediate computation of interval estimators for active substance and comparator.

All interval estimators yielded CIs with acceptable coverage when sample sizes exceeded 15, except for the exact Clopper‐Pearson intervals (Figure 1). When comparing the coverage and divergence from the desired coverage across all scenarios, we found that the MiettinenvNurminen score (coverage 0.951) and Baptista–Pike mid‐*p* interval (coverage 0.955) provided more accurate coverage than the other methods. The largest divergence was observed for the Wald method based on Clopper–Pearson intervals (coverage 0.979) (Table 1). We observed similar patterns when analyzing each scenario separately (Table S1).

**TABLE 1:** Divergence from the desired 0.95 coverage for each estimator, coverage, and Monte–Carlo standard error across all combinations of sample size, sequence ratio, and comparator sequence ratio.

Estimator	Divergence	Coverage	MCSE
Miettinen–Nurminen	0.001	0.951	< 0.001
Baptista–Pike mid‐*p*	0.005	0.955	< 0.001
Jeffrey's sum	0.006	0.944	< 0.001
Agresti–Coull sum	0.007	0.957	< 0.001
Woolf logit	0.007	0.957	< 0.001
Clopper–Pearson sum	0.029	0.979	< 0.001

Abbreviation: MCSE, Monte–Carlo standard error.

## 4 Conclusions

In this simulation study of a sequence symmetry analysis, we found that the 95% CIs for the active comparator SR based on the Miettinen–Nurminen score estimator provided the most accurate coverage. The previously recommended methods for estimating CIs or active comparator SRs assume that the variance of the SR for each separate drug is normally distributed, which is not always the case. The Miettunen‐Nurminen intervals are less conservative, especially when the total number of sequences is low. The previously proposed method of deriving CIs from the sum of the variances of the component SRs was clearly inferior, regardless of the interval estimator used for the individual SRs. We recommend the use of the Miettinen‐Nurminen score estimator for future active comparator SSA studies to ensure the estimation of CIs with the desired coverage. This ensures that SRs are classified correctly according to statistical significance, which is common practice in hypothesis‐generating studies.

## Conflicts of Interest

Martin Torp Rahbek and Lars Christian Lund report no conflicts of interest. Jesper Hallas reports participation in post‐authorization safety studies funded by Novo Nordisk with money paid to his employer and with no personal fees involved.

## Supporting information


**Data S1.** Supporting Information.

## Appendix 1

$$\mathrm{Var}\left(\ln\left(\mathrm{SR}\right)\right)={\left(\ln\left(\frac{\text{Upper bound of the}\;95\%\mathrm{CI}}{\text{Lower bound of the}\;95\%\mathrm{CI}}\right)÷3.92\right)}^{2}$$

where SR = sequence ratio, CI = compatibility interval.

## Appendix 2

$$95\%\mathrm{CI}=\exp\left(\ln\left(\frac{{\mathrm{SR}}_{1}}{{\mathrm{SR}}_{2}}\right)\pm 1.96\cdot \sqrt{\mathrm{Var}\left(\ln\left({\mathrm{SR}}_{1}\right)\right)+\mathrm{Var}\left(\ln\left({\mathrm{SR}}_{2}\right)\right)}\right)$$

where SR = sequence ratio, CI = compatibility interval.

## Appendix 3

$$\mathrm{SE}=\sqrt{\frac{1}{a}+\frac{1}{b}+\frac{1}{c}+\frac{1}{d}}$$

where *a*, *b*, *c*, and *d* correspond to the number of exposure‐to‐outcome and outcome‐to‐exposure sequences observed for the drug of interest and the comparator drug. SE = standard error.
