# Supplementary material for: Obtaining Valid Compatibility Intervals for Sequence Symmetry Analyses Utilizing Active Comparators: A Simulation Study
Source: Pharmacoepidemiol Drug Saf. 2025 May 20;34(6):e70160. doi: 10.1002/pds.70160 (PMC12092166; doi:10.1002/pds.70160)
Supplement: Supplementary file 1 — Data S1. Supporting Information. [file PDS-34-e70160-s001.docx]

Supplementary table 1:

Obtaining valid compatibility intervals for sequence symmetry analyses utilizing active comparators: A simulation study

Martin Torp Rahbek (ORCID 0000-0001-7506-3426)

Jesper Hallas (ORCID 0000-0002-8097-8708)

Lars Christian Lund (ORCID0000-0001-8651-6072)

Clinical Pharmacology, Pharmacy and Environmental Medicine, University of Southern Denmark, Denmark

**Corresponding author**
Martin Torp Rahbek
Clinical Pharmacology, Pharmacy and Environmental Medicine
University of Southern Denmark
Campusvej 55
5230 Odense M

[mrahbek@health.sdu.dk](mailto:mrahbek@health.sdu.dk)

**Supplementary table 1** Divergence from the desired 0.95 coverage, coverage and Monte Carlo standard error across all replications for each combination of estimator, sequence ratio and comparator sequence ratio

| **SR** | **cSR** | **Estimator** | **Divergence** | **Coverage** | **MCSE** |
| --- | --- | --- | --- | --- | --- |
| SR=0.5 | cSR=0.5 | Miettinen-Nurminen | 0.002 | 0.952 | <0.001 |
|  |  | Jeffrey's | 0.007 | 0.943 | <0.001 |
|  |  | Baptista-Pike mid-p | 0.008 | 0.958 | <0.001 |
|  |  | Woolf logit | 0.010 | 0.960 | <0.001 |
|  |  | Agresti-Coull | 0.012 | 0.962 | <0.001 |
|  |  | Clopper-Pearson | 0.032 | 0.982 | <0.001 |
|  |  |  |  |  |  |
| SR=0.5 | cSR=1 | Miettinen-Nurminen | 0.001 | 0.949 | <0.001 |
|  |  | Baptista-Pike mid-p | 0.002 | 0.952 | <0.001 |
|  |  | Woolf logit | 0.005 | 0.955 | <0.001 |
|  |  | Agresti-Coull | 0.005 | 0.955 | <0.001 |
|  |  | Jeffrey's | 0.007 | 0.943 | <0.001 |
|  |  | Clopper-Pearson | 0.029 | 0.979 | <0.001 |
|  |  |  |  |  |  |
| SR=0.5 | cSR=2 | Miettinen-Nurminen | 0.001 | 0.951 | <0.001 |
|  |  | Baptista-Pike mid-p | 0.003 | 0.953 | <0.001 |
|  |  | Agresti-Coull | 0.005 | 0.955 | <0.001 |
|  |  | Woolf logit | 0.007 | 0.957 | <0.001 |
|  |  | Jeffrey's | 0.010 | 0.940 | <0.001 |
|  |  | Clopper-Pearson | 0.028 | 0.978 | <0.001 |
|  |  |  |  |  |  |
| SR=1 | cSR=1 | Jeffrey's | 0.000 | 0.950 | <0.001 |
|  |  | Miettinen-Nurminen | 0.001 | 0.951 | <0.001 |
|  |  | Agresti-Coull | 0.002 | 0.952 | <0.001 |
|  |  | Woolf logit | 0.005 | 0.955 | <0.001 |
|  |  | Baptista-Pike mid-p | 0.007 | 0.957 | <0.001 |
|  |  | Clopper-Pearson | 0.027 | 0.977 | <0.001 |
|  |  |  |  |  |  |
| SR=1 | cSR=2 | Miettinen-Nurminen | 0.001 | 0.949 | <0.001 |
|  |  | Baptista-Pike mid-p | 0.003 | 0.953 | <0.001 |
|  |  | Woolf logit | 0.005 | 0.955 | <0.001 |
|  |  | Jeffrey's | 0.006 | 0.944 | <0.001 |
|  |  | Agresti-Coull | 0.006 | 0.956 | <0.001 |
|  |  | Clopper-Pearson | 0.029 | 0.979 | <0.001 |
|  |  |  |  |  |  |
| SR=2 | cSR=2 | Miettinen-Nurminen | 0.002 | 0.952 | <0.001 |
|  |  | Jeffrey's | 0.006 | 0.944 | <0.001 |
|  |  | Baptista-Pike mid-p | 0.008 | 0.958 | <0.001 |
|  |  | Woolf logit | 0.010 | 0.960 | <0.001 |
|  |  | Agresti-Coull | 0.011 | 0.961 | <0.001 |
|  |  | Clopper-Pearson | 0.031 | 0.981 | <0.001 |

SR = Sequence ratio, cSR = comparator sequence ratio, MCSE = Monte carlo standard error
